# Metabolomics profiling in acute liver transplant rejection in a pediatric population

**DOI:** 10.1038/s41598-022-18957-4

**Published:** 2022-11-04

**Authors:** Jennifer K. Frediani, Yara S. Beyh, Nitika Gupta, Adrianna L. Westbrook, Rebecca Cleeton, Maria Cordero, Albert Hernandez, ViLinh Tran, Dean P. Jones, Miriam B. Vos

**Affiliations:** 1 Nell Hodgson Woodruff School of Nursing, Emory University, Atlanta, GA USA; 2 Nutrition and Health Sciences, Rollins School of Public Health, Laney Graduate School, Emory University, Atlanta, GA USA; 3 Department of Pediatrics, Emory University School of Medicine, Atlanta, GA USA; 4 Transplant Services, Children’s Healthcare of Atlanta, Atlanta, GA USA; 5 Department of Pulmonology, Emory University School of Medicine, Atlanta, GA USA

**Keywords:** Biochemistry, Medical research

## Abstract

Pediatric liver transplantation rejection affects 20% of children. Currently, liver biopsy, expensive and invasive, is the best method of diagnosis. Discovery and validation of clinical biomarkers from blood or other biospecimens would improve clinical care. For this study, stored plasma samples were utilized from two cross-sectional cohorts of liver transplant patients at Children’s Healthcare of Atlanta. High resolution metabolic profiling was completed using established methods. Children with (n = 18) or without (n = 25) acute cellular rejection were included in the analysis (n = 43 total). The mean age of these racially diverse cohorts ranged from 12.6 years in the rejection group and 13.6 years in the no rejection group. Linear regression provided 510 significantly differentiating metabolites between groups, and OPLS-DA showed 145 metabolites with VIP > 2. A total of 95 overlapping significant metabolites between OPLS-DA and linear regression analyses were detected. Pathway analysis (p < 0.05) showed bile acid biosynthesis and tryptophan metabolism as the top two differentiating pathways. Network analysis also identified tryptophan and clustered with liver enzymes and steroid use. We conclude metabolic profiling of plasma from children with acute liver transplant rejection demonstrates > 500 significant metabolites. This result suggests that development of a non-invasive biomarker-based test is possible for rejection screening.

## Introduction

According to the United Network for Organ Sharing (UNOS), there were 8906 liver transplants in 2020 across the United States (US), which marks a 0.1% increased from 2019^1^. To date, there are 18,596 new addition to the waiting list for liver transplant among candidates under the age of 18 years, with the majority of 6827 candidates being among children under 1 year old^2^. On the other hand, there has been 18,310 liver transplantations done in children under the age of 18 years, with the majority of 6879 operations being between the ages of 1 and 5 years old^2^. Graft failure was observed in 6.5% of the cases after 6 months and 6.5% of the cases 1-year post-transplant for transplants performed in 2018^3^.

One major concern of liver transplant, as with any other organ transplantation, is the possibility of allograft rejection. Specifically, in the pediatric population, there is a 60% chance of acute cellular rejection (ACR) over the first 5 years^4–6^. Rejection is defined as the immunological attack by the host on the graft encompassing both acute and long-term inflammatory changes^7^. ACR is known to occur anytime, but most frequently within the first-year post-transplant^8^. ACR is diagnosed by finding at least two of the three features: portal inflammation, bile duct injury, and venous endotheliolitis on histopathology^7,9^.

Liver biopsy remains the gold standard technique to detect rejection^7,8^. Liver biopsy is an invasive technique and requires a surgical procedure to extract a sample of the organ. ACR is defined by the following histologic features including (1) predominantly mononuclear portal inflammation (2) sub-endothelial inflammation, and (3) lymphocytic cholangitis^8^. ACR causes significant morbidity and is one of the leading causes of liver graft loss in children^10^. Thus, better understanding of the mechanisms underlying ACR and non-invasive biomarkers are needed. Therefore, we aimed to examine metabolic pathways to characterize pediatric liver transplant rejection using ultra-high-resolution metabolomics (HRM).

Metabolomics is the study of small molecule metabolites in biofluids and tissues to identify biomarkers associated with altered metabolic pathways, which allows better understanding of downstream effects of genes and proteins^5,11^. Metabolites are modulated by proteins and other enzymatic functions, hence increasing their sensitivity to biological stressors, and thus their ability to reflect the disease-induced alterations, as well as the functional phenotype of the organism^5,11^. Despite the poor understanding of how metabolic changes influence ACR, metabolomics has allowed the discovery of several endogenous pathophysiological metabolites in the liver involved in the rejection of the organ post-transplantation^11–15^.

## Results

### Study participants

A total of 43 children, equally distributed among males and females, were included. Demographics and characteristics are summarized in Table 1. Children were divided in two groups, those with ACR (n = 18) and no ACR (n = 25). The average age was 13.2 years, with the ACR group being fairly age matched with the no ACR group, 12.6 and 13.6 years, respectively (non-significant). The ethnic and racial variability of the participants included white (57.1%), black (28.6%) and Hispanic (11.9%) which was also representative of our transplant population cohort. The most frequent diagnosis across no ACR group was biliary atresia (32.0%), whereas the most common diagnosis among those with ACR was AIH (29.4%). The ACR group was on average 1027 days post-transplant when recruited, while the no-ACR group was on average 2936 days post-transplant (p-value = 0.0004). Half of the children were taking steroid treatment, of which 88.2% were among those with ACR but only 24% among no ACR participants (p-value < 0.0001). The main biochemical characteristics differentiating the two groups included gamma glutamyltransferase (GGT) with a mean difference between the groups of 311.2 (p-value = 0.0015), albumin with a mean difference of 0.38 (p-value = 0.03), and days from transplant with a mean difference of 1909.9 days (p-value = 0.0004).

**Table 1:** Participant demographics by rejection status.

Characteristic mean (SD) N (%)	Rejection (n = 18)	No rejection (n = 25)	Total (n = 43)	p-value
Female	9 (53%)	12 (48%)	21 (50%)	0.753
Age (years)	12.6 (7.0)	13.6 (4.2)	13.2 (5.5)	0.614
**Race/ethnicity**				0.218
Black	7 (41%)	5 (20%)	12 (29%)	
White	8 (47%)	16 (64%)	24 (57%)	
Hispanic	1 (6%)	4 (16%)	5 (12%)	
**Diagnosis**				
Biliary atresia	2 (12%)	8 (32%)	10 (24%)	
AIH	5 (29%)	0 (0%)	5 (12%)	
ALF	2 (12%)	8 (32%)	10 (24%)	
Urea cycle defect	3 (18%)	0 (0%)	3 (7%)	
Hepatoblastoma	2 (12%)	0 (0%)	2 (5%)	
ALT (units/L)	300 (346)	113 (278)	189 (317)	0.074
AST (units/L)	224 (194)	92.6 (226)	146 (221)	0.051
GGT	419.2 (321.5)	108 (193.1)	236.9 (291.75)	0.0015**
Hemoglobin	12.17 (1.85)	13.07 (1.81)	12.73 (1.88)	0.13
Total bilirubin	2.43 (3.28)	0.8 (0.54)	1.43 (2.19)	0.06
Albumin	3.52 (0.74)	3.91 (0.35)	3.77 (0.55)	0.03*
Days from transplant	1030 (1350)	2940 (1820)	2160 (1890)	0.0004**
**Steroid use**	15 (88%)	6 (24%)	21 (50%)	< 0.0001***

SD, standard deviation; AIH, autoimmune hepatitis; ALF, acute liver failure; ALT, alanine aminotransferase; AST, aspartate aminotransferase; GGT, gamma glutamyltransferase.

*p-value < 0.05; **p-value < 0.001; ***p-value < 0.0001.

### Metabolic signatures in plasma

**Figure 1:**
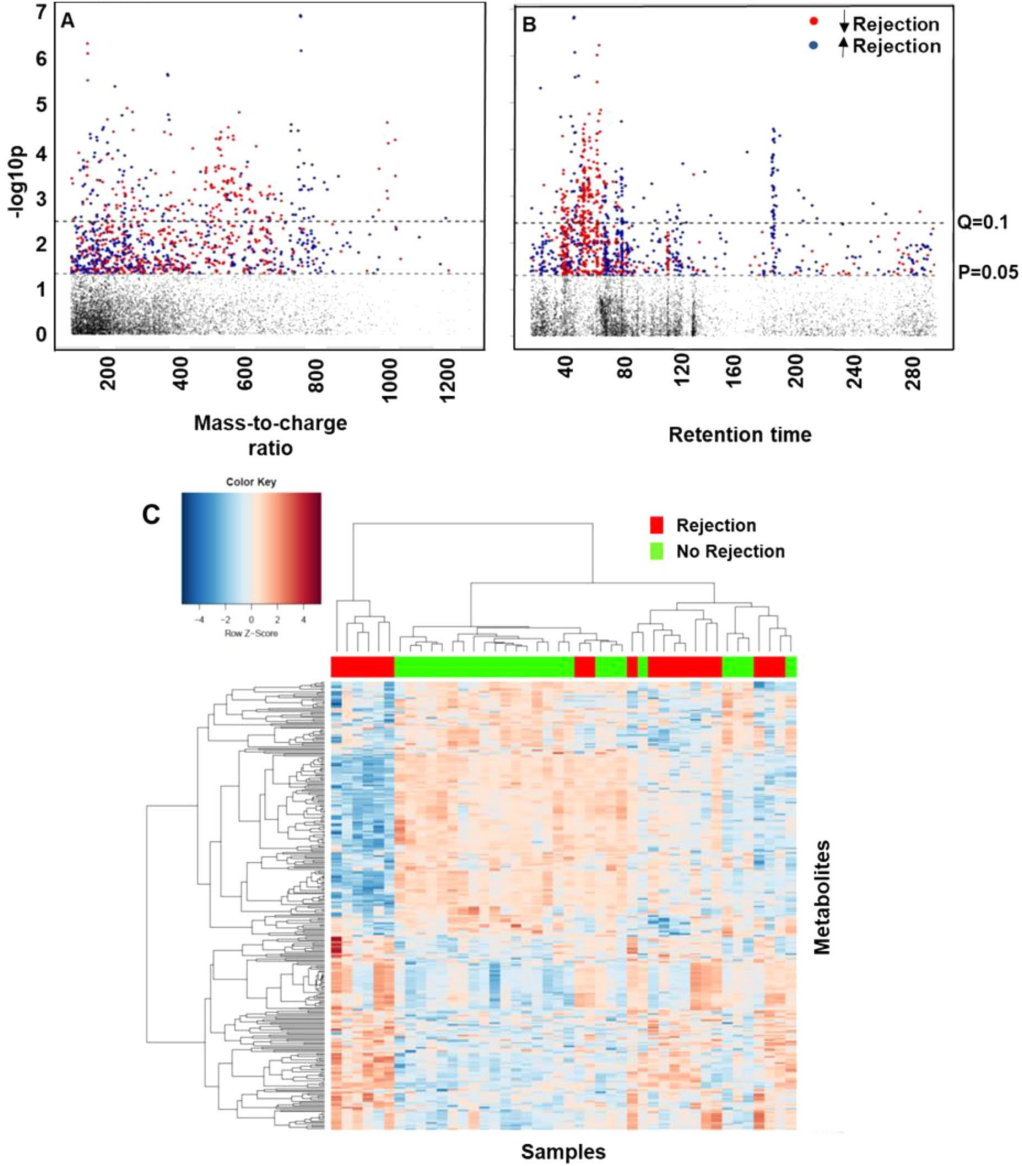
Data visualization: (**A**) and (**B**) are Manhattan Plots derived from the linear regression. The red dots are lower within the rejection group, while the blue dots are higher. On the y-axis –log 10p, (**A**) represents Type I Manhattan plot with x-axis showing the *m/z*; whereas (**B**) represents type II Manhattan plot with x-axis showing the retention time (s). A significant m/z was determined for 292 metabolites at q = 0.1. (**C**) is atwo-way Hierarchical Cluster Analysis derived from the linear regression using the *m/z* of all the significant features. Red = Rejection, Green = No rejection. The y-axis represents the *m/z*, while the x-axis represents the samples.

The Manhattan plots in Fig. 1A,B show differences between detected metabolites in ACR and no ACR groups, the red dots represent down-regulated metabolites in the ACR group and the blue dots upregulated metabolites. Panel A, with the mass-to-charge ratio on the x-axis, clearly shows a significant difference in the spread between the majority of the significant (q < 0.1) red and blue dots. As for panel B, with the retention time on the x-axis, the significant red dots tend to have a low retention time (40–80 s), while the significant blue dots have a higher retention time (~ 200 s). The two panels imply that large molecules, such as vitamins, amino acids and bile acids, tend to be increased in the rejection group. Furthermore, the two-way hierarchal cluster analysis, presented in Fig. 1C, showed a distinction between the two groups, the ACR group shown in red and the no ACR group in green.

**Figure 2:**
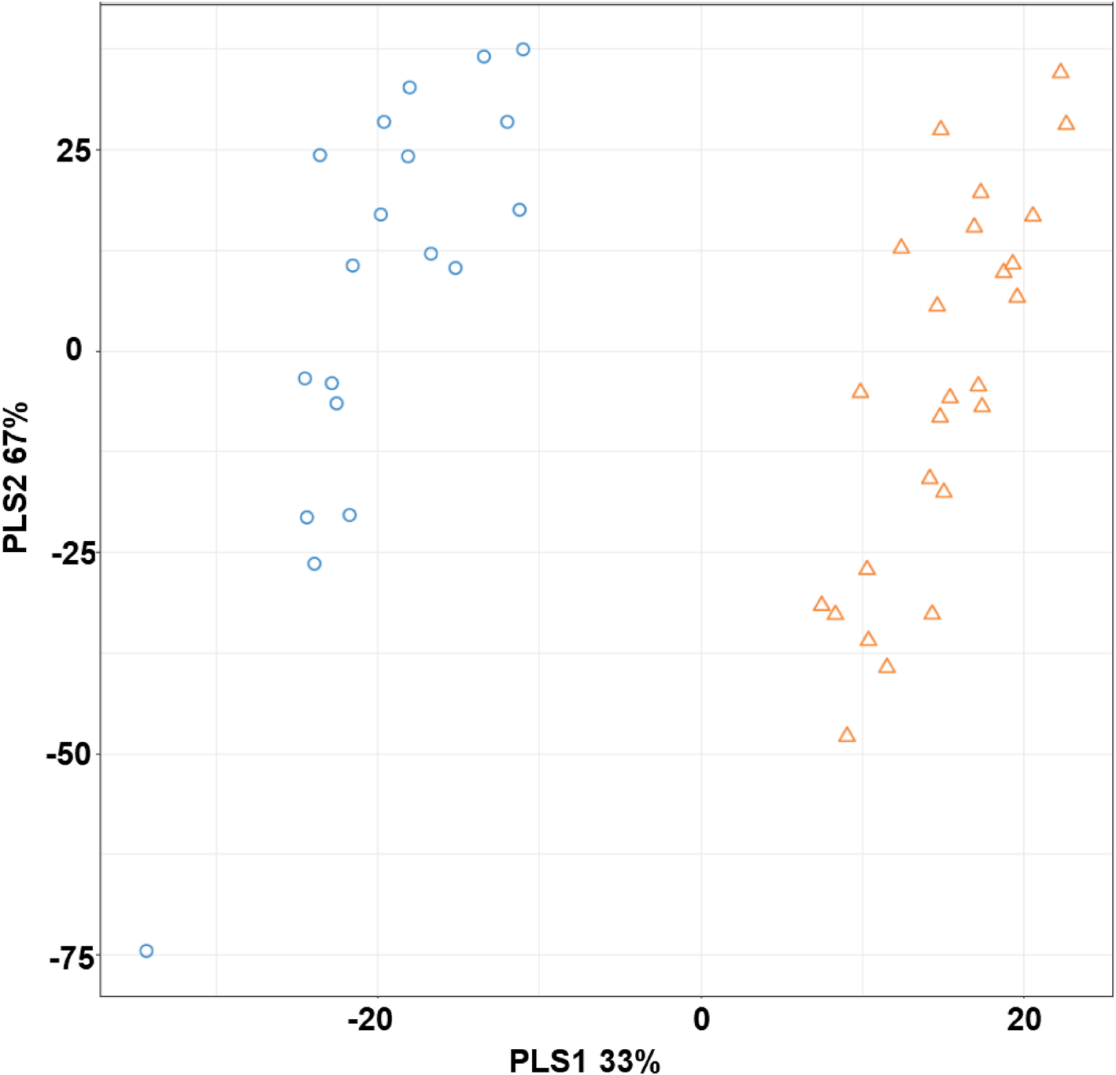
OPLS-DA plot using the *m/z* results of the significant metabolites with VIP > 2. Blue = Rejection, Orange = No rejection. Component 1 on the x-axis represents 33% of the significant features; while Component 2 on the y-axis represents 67% of the samples.

**Figure 3:**
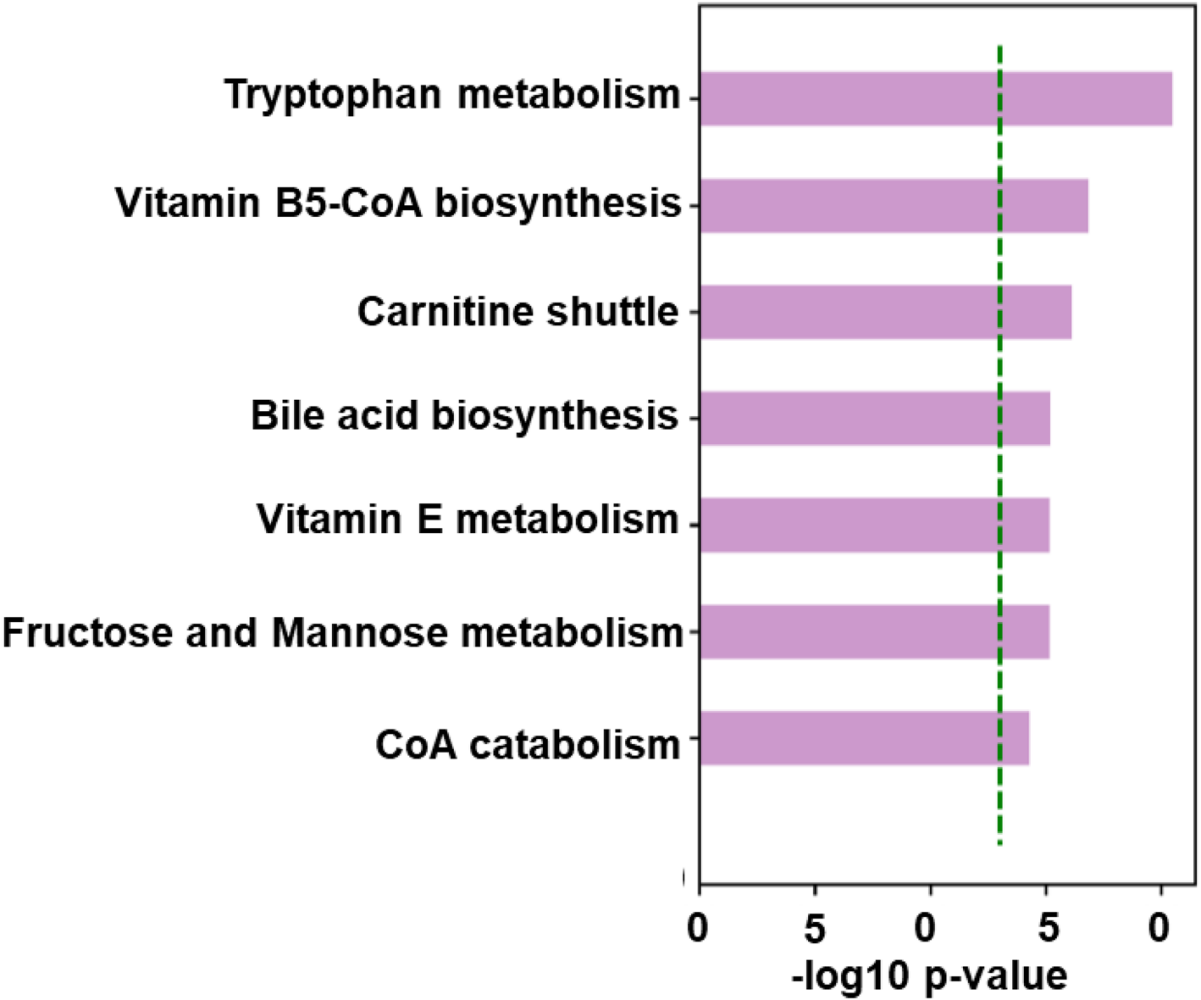
Pathway Analysis using significant metabolites observed in the linear regression (raw p < 0.5).

To investigate the metabolic difference between ACR and no ACR, we compared the two groups using two different methods. First, by linear regression and controlling for the days from transplant before the biopsy and then using the OPLS-DA. Linear regression detected 510 significantly differentiating metabolites between ACR and no ACR. The OPLS-DA showed 145 metabolites with a VIP > 2. The supervised distinction between ACR and no ACR groups is shown in Fig. 2, where the blue circles are the ACR group, and the orange triangles represent the no ACR group. The results of the fivefold cross-validation produced a mean accuracy of 0.9667 (standard deviation = 0.0745). The outcome resulted in 95 overlapping significant metabolites between OPLS-DA and linear regression analyses. We did find significant associations between the certain phenotypes such as steroid use, GGT, and time since transplant and PLS component scores which may have influenced the differential results between rejection and no rejection as shown in Table S1. The significant pathways obtained from the linear regression analysis included tryptophan metabolism, vitamin B5-CoA biosynthesis, carnitine shuttle, bile acid biosynthesis, vitamin E metabolism, fructose and mannose metabolism, and CoA metabolism. The annotated significant metabolites for vitamin B5-CoA biosynthesis, carnitine shuttle, vitamin E metabolism, fructose and mannose metabolism, and CoA metabolism can be found in Table 2. The top two differentiating pathways (p < 0.05) were bile acid biosynthesis and tryptophan metabolism. Based on the linear regression analysis, we detected 30 metabolites, of which 9 were significant in the bile acid biosynthesis pathway. The tryptophan metabolism pathway showed 46 metabolites, of which 11 were significant. The pathway analysis results are summarized in Fig. 3.

**Table 2:** Significant metabolites identified in the other five significant pathways leading to liver transplantation rejection.

Metabolite	*m/z*	Retention time	Identification level
**Vitamin B5—CoA biosynthesis from pantothenate**			
l-Cysteine	122.027	187.5	2
Unknown	323.082	84.7	–
Unknown	180.055	109.7	–
**Carnitine shuttle**			
Gamma-linolenyl-carnitine	422.326	43.3	3
Palmitoyl carnitine	400.341	42.0	3
Steroylcarnitine	428.372	41.8	2
Unknown	468.388	42.7	–
Heptadecanoyl carnitine	414.357	42.2	2
Pentadecenoyl coenzyme A	992.344	69.3	2
**Fructose and mannose biosynthesis**			
l-Galactose	181.072	228.2	2
l-Iditol	184.085	74.6	3
Fructose 1-phosphate	261.039	258.5	2
**Vitamin E metabolism**			
Oleoylcarnitine	427.360	42.4	3
13-hydroxy-alpha-tocopherol	429.372	41.7	3
13-hydroxy-alpha-tocopherol	447.383	41.3	3
4alpha-Carboxy-5alpha-cholesta-8-en-3beta-ol	431.351	41.4	2
Barringtogenol C	491.373	41.0	3
**CoA catabolism**			
Unknown	339.076	84.2	–
Unknown	178.040	111.8	–

**Figure 4:**
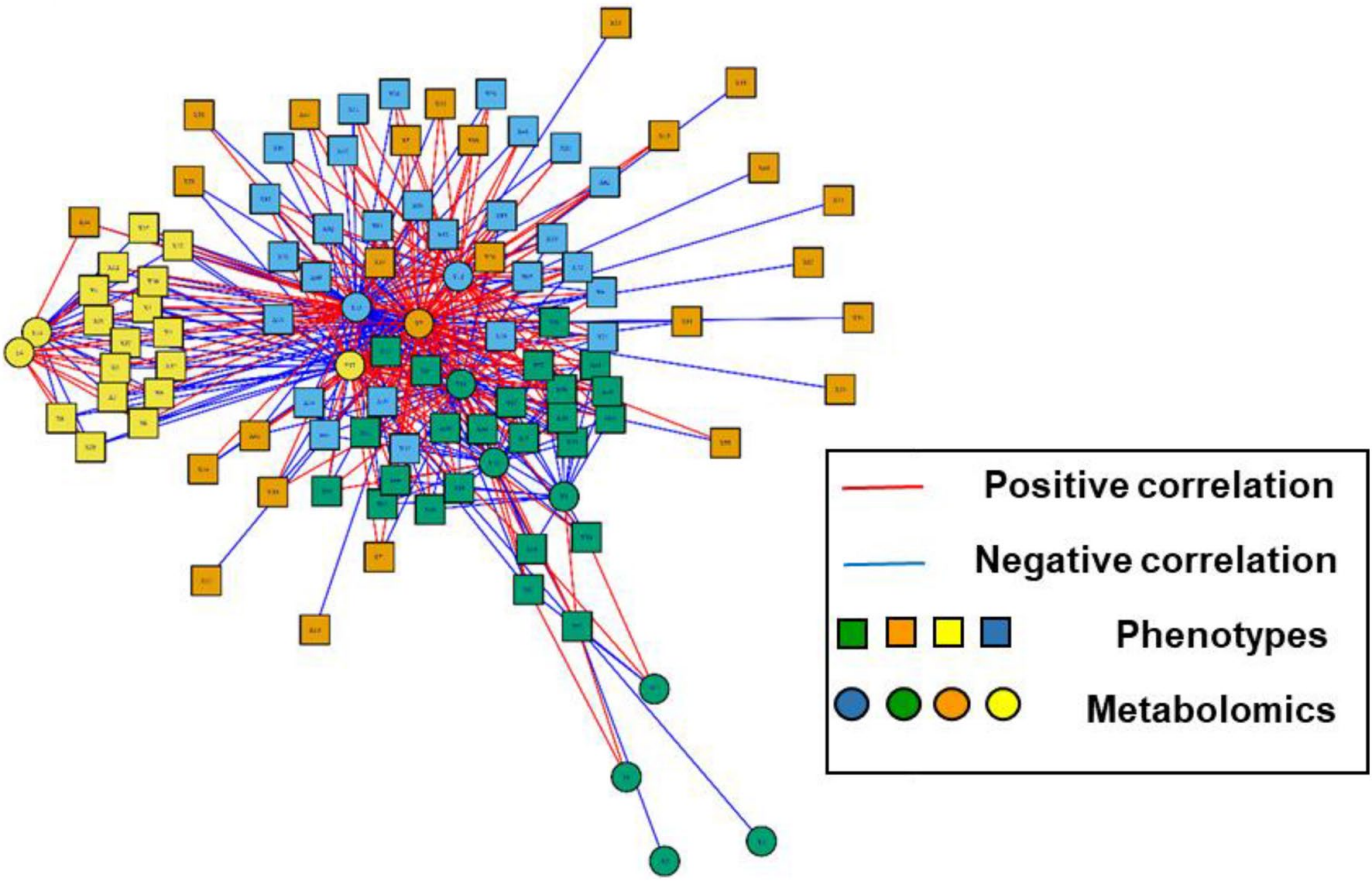
Multidata network threshold showing the four distinct clusters; red edges: positive correlation, blue edges: negative correlation; square nodes: metabolites, circle nodes: phenotypes.

The xMWAS network analysis using significant metabolites from linear regression analysis produced four distinct communities. Focusing on the most significant pathways obtained earlier, clusters 2 and 4 are of interest. Cluster 2 included tryptophan (m/z 205.0972; Schymanski Level 1; decreased in ACR) and glycocholate (m/z 466.3163; Schymanski Level 1; increased in ACR), which correlated with all liver enzymes and steroid use. Further, cluster 4 included glucosamine (m/z 180.0867; Schymanski Level 1; increased in ACR) and methyl-indole-3-acetate (m/z 190.0863; decreased in ACR), which correlated with body mass index and ACR status. Figure 4 shows the four different clusters obtained. Further, Table 3 shows the top 12 significant metabolites that were overlapping between the linear regression and the OPLS-DA, with their respective confidence, *m/z*, retention time, compound name, class, and the corresponding adduct.

**Table 3:** Top 12 significant metabolites overlapping between days from transplant analysis and OPLS-DA.

Confidence	*m/z*	Time	Compound name	Class	Adduct
3	141.53247	189	l-Cystine	Carboxylic acid	M + ACN + 2H
3	142.53045	184.3	Inositol cyclic phosphate	Organic phosphoric acid	M + ACN + 2H
3	162.04574	185.3	l-Cystine	Carboxylic acid	M + 2ACN + 2H
3	518.32187	54.3	LysoPC(18:3(6Z9Z12Z))	Glycerophospholipid	M + H
3	524.37101	53.5	LysoPC(18:0)	Glycerophospholipid	M + H
3	525.37435	53.5	LysoPC(0:018:0)	Glycerophospholipid	M + H_[+ 1]
3	526.37749	53.6	LysoPC(18:0)	Glycerophospholipid	M + H_[+ 2]
3	547.35789	53.4	Hovenidulcigenin B	Prenol lipids	M + H
	206.98202	57	Unknown		
	536.29162	57.6	Unknown		
	562.32644	57.7	Unknown		
	563.32978	57.7	Unknown		

## Discussion

We aimed to examine metabolic pathways to characterize pediatric liver transplant rejection using ultra-high-resolution metabolomics. From our analyses, we were able to determine distinct metabolites and metabolic pathways that may differentiate between those in rejection and those who were not.

Our analyses detected 11 significant metabolites involved in tryptophan metabolism through pathway analysis (Table 2), which is in line with other studies. Previous research involving tryptophan metabolism as a significant metabolite hypothesized that rejection-specific pathways are linked to immune T cells modulation and the role of nutrients in their functioning^16,17^. This could be important as the link between T cells and other nutrients is a two-way street, where the activation of T cells requires the metabolism of other molecules, mainly amino acids such as leucine, and the flux of glucose, lactate, lipids, proteins, nucleic acids and carbohydrates requires activated T cells^18,19^. Furthermore, the role of immune T cells is also linked to the metabolism of the amino acid tryptophan, which is one of the two prominent pathways we distinguished. In fact, the degradation of tryptophan to N-formyl kynurenine, converted later to niacin (vitamin B3), is involved in the activation of the Th1-type cytokine interferon-γ (IFN-γ), which could have implications on the graft itself or on the immune response exerted by the body on the activation of the immune T cells^20,21^. This could be related to the fact that kynurenines can highly inhibit the proliferation of T-cells using the indoleamine 2, 3, dioxgenase (IDO) mechanism^21^. On the other hand, the role infiltration of Tregs is essential to minimalize the side effects that could arise in the newly transplanted organ by inducing a short-term benign inflammation^17^. Furthermore, it was observed that kynurenine levels were altered in ACR compared to those without ACR by an increased activity of the kynurenine/tryptophan pathways^22^. Kynurenine is known to be an intermediate and rate limiting step in tryptophan metabolism, in addition to its implication in cellular stress mechanisms and inflammatory responses. This step is catalyzed by IDO and tryptophan 2–3 deoxygenase, with the latter being liver-specific^22^.

As for the other significantly differentiating metabolite, our analyses detected 9 significant metabolites involved in bile acid synthesis (Table 2). These included increased levels of bile acids such as taurine and glycine-conjugates, which can act as signaling molecules or ligands of the farnesoid X receptor (FXR) that controls the expression of genes involved in lipids, lipoproteins and glucose metabolism, justifying its role as a liver functionality biomarker^11^. Bile acid synthesis serves as a significant contributor in understanding the hepatic biochemical pathways associated with liver transplantation rejection^5,11,12,16^. In fact, an accumulation of bile acids and any of its metabolites may affect the recovery of bile flow in graft patients^11^. This could be linked to an alteration of a number of genes related to bile acid synthesis and transport such as BAAT, CYP7A1, BSEP and to nuclear factors acting in their regulation (HNFa, FXR, SREBF1). Bile acids have been reported to activate the JNK1/2 pathway mainly in the hepatocytes by direct and indirect mechanisms, which was linked to the downregulation of CYP7A1 and bile acid synthesis^23^. Furthermore, to activate JNK 1/2, there is an apparent need to produce ceramide by activating acidic sphingomyelinase, which is also activated by TNF-a in some hepatocyte locations. Bile acids have been linked to the activation of the G-protein coupled receptor TGR5, which is involved in hepatoprotection as well as energy production. The latter is linked to the activation of the AKT and ERK 1/2 signaling pathways in hepatocytes^23^. This is possible because the enzymes needed for the synthesis of bile acids are found in various locations in the hepatocytes such as the smooth endoplasmic reticulum, mitochondria, peroxisomes and cytoplasm. However, it remains unclear how the intermediates can move from one location to the other. Other studies have shown that the main bile acid presented is conjugated with taurine (TCA), leading to increased cytotoxicity from bile acid post-transplant^30^. These results corroborated with NMR studies showing a low recipient/donor tauro-conjugated bile aid ratio in the first week post-transplant, which is associated with a higher risk of rejection^11,23^. Furthermore, it has been shown that bile acid, in ACR, has the capacity to reduce the activity of certain genes, such as CYP7A1, involved in bile acid synthesis^24^.

Lastly, the xMWAS clusters analysis we conducted provided a framework for integrative analysis and differentiating network analysis. Figure 4 shows identification and visualization of the association between various metabolites mentioned earlier and possible phenotypes that could lead to ACR (steroid use, ALT, AST, days from transplant, etc.). Cluster number 2 is the most important as it connects the top two significant metabolites in our analysis with other variables, most significantly the rejection of the transplantation. The concentration of the metabolites in this cluster validates the results we obtained through linear regression and OPLS-DA, that tryptophan metabolism and bile acid biosynthesis are the top two significant metabolites in ACR in a pediatric population. Furthermore, it demonstrates an association with first, liver enzymes, which are often used as an initial proxy of liver disease and second, steroid use, which in our study was significantly higher in those with liver rejection. These clinically expected associations provide support for the novel finding of the clustering of tryptophan metabolism and bile acid biosynthesis alterations with rejection.

Finally, the conducted analysis showed a clear clustering and separation of ACR and no ACR groups. The hierarchal cluster in Fig. 1, reflects the existence of several groups of metabolites involved in ACR, which are different from those of the no ACR group. Further, Fig. 4 shows a segregation of variables into four clusters with various phenotypes. Future investigations should approach this population from a patient perspective to segment into possible phenotypic subtypes that may lead to prediction of risk or severity of rejection. We can conclude that metabolic profiling has the power to detect changes that go unnoticed with morphological or clinical markers. Thus, suggesting a power to predict liver graft function pre-implantation. The use of this approach could help in making decisions about accepting or rejecting organs and maximizing graft survival.

To the best of our knowledge, this study was one of the first to evaluate the metabolic pathways related to liver transplantation rejection in a pediatric population, as it was challenging to find pediatric metabolomics studies in the literature. Through this, we will start a series of studies directed towards the importance of metabolomics in the determination of transplantation rejection or success and reducing the need for invasive techniques such as biopsies. However, one of the limitations was the inability to compare our findings to other available studies in the pediatric population, and we had to refer to studies in the adult population. Furthermore, we had a relatively small sample size (n = 43), nevertheless, we were able to examine the significantly differentiating pathways involved in liver transplantation rejection using a stringent q value. Finally, there were several univariate differences between the two groups in this pilot convenient sample. In the future, we will recruit a clean sample and match on steroid use, time since transplant and liver injury severity.

## Methods

### Sample collection

For this study, patients were drawn from two simultaneously recruiting studies in order to capture both stable liver transplant patients and those presenting for a liver biopsy for possible rejection. Both studies were approved by the Emory University Institutional Review Board and in line with all research guidelines. Inclusion criteria were (1) post liver transplant, (2) plasma sample available and (3) either rejection or no rejection by medical record review at the time of the plasma sample collection. We included 43 plasma samples from two studies. Study 1 was the *Evaluation of cardiovascular risk markers in pediatric transplant recipients* study (PI Vos; IRB#00076255). Patients, ages 10–21 years old, who had undergone a liver transplant at least 12 months prior and who did not have ACR within the last 3 months were included in this study. Study 2 was the *Serum markers and magnetic resonance imaging (MRI) in the evaluation of liver disease* study (PI Vos, IRB#00002117 and 00094514). All participants were assented, and informed consent was obtained from parents/guardians to participate. This study enrolled any child scheduled for liver biopsy, without fever (in prior 2 weeks) and no chronic renal disease or insufficiency. ACR was determined by pathology and no ACR was determined by medical chart review for any rise in liver enzymes resulting in a clinical rejection diagnosis or liver biopsy within 1 month prior to or after the research visit. Venous blood draws were completed between 08/2014–02/2018 at time of liver biopsy using ethylenediaminetetracetic acid (EDTA) blood tubes for collection of plasma. Demographic questionnaires were also collected at this time. EDTA tubes were inverted several times and immediately put on ice. Plasma was centrifuged for 10–20 min at 1200–1300 rcf, aliquoted and immediately frozen at − 80 °C.

### HRM methods

HRM was completed using established methods^25,26^. Plasma samples were prepared and analyzed in batches of 20; each batch included duplicate analysis of pooled human plasma (QStd-3) for quality control purposes and reference standardization. Prior to analysis, plasma aliquots were removed from storage at − 80 °C and thawed on ice. Each cryotube is then vortexed briefly to ensure homogeneity, and 50 μL transferred to a clean microfuge tube. Immediately after, plasma was treated with 100 μL of ice-cold LC–MS grade acetonitrile (Sigma Aldrich) containing 2.5 μL of internal standard solution with eight stable isotopic chemicals selected to cover a range of chemical properties. Following addition of acetonitrile, plasma is then equilibrated for 30 min on ice, upon which precipitated proteins are removed by centrifuge (16.1×*g* at 4 °C for 10 min). The resulting supernatant (100 μL) is removed, added to a low volume autosampler vial and maintained at 4 °C until analysis (< 22 h).

Samples were analyzed in triplicate using 10 μL injections and separate HILIC and C_18_ chromatography columns with detection by high-resolution mass spectrometry (Q-Exactive HF Orbitrap, Thermo Scientific, San Jose, CA). During HILIC chromatography, the electrospray ionization (ESI) source is operated in positive ion mode while the reverse phase column is flushing with wash solution. Flow rate is maintained at 0.35 mL/min until 1.5 min, increased to 0.4 mL/min at 4 min and held for 1 min. Solvent A is 100% LC–MS grade water, solvent B is 100% LC–MS grade acetonitrile and solvent C is 2% formic acid (v/v) in LC–MS grade water. Initial mobile phase conditions are 22.5% A, 75% B, 2.5% C hold for 1.5 min, with linear gradient to 77.5% A, 20% B, 2.5% C at 4 min, hold for 1 min, resulting in a total analytical run time of 5 min. During the flushing phase, the HILIC column is equilibrated with a wash solution of 77.5% A, 20% B, 2.5% C.

The C_18_ column is operated parallel to the HILIC column. During operation of the C_18_ method, the ESI source is operated in negative ion mode while the HILIC column is flushing with wash solution. Flow rate is maintained at 0.4 mL/min until 1.5 min, increased to 0.5 mL/min at 2 min and held for 3 min. Solvent A is 100% LC–MS grade water, solvent B is 100% LC–MS grade acetonitrile and solvent C is 10 mM ammonium acetate in LC–MS grade water. Initial mobile phase conditions are 60% A, 35% B, 5% C hold for 0.5 min, with linear gradient to 0% A, 95% B, 5% C at 1.5 min, hold for 3.5 min, resulting in a total analytical run time of 5 min. During the flushing phase (HILIC analytical separation), the C_18_ column is equilibrated with a wash solution of 0% A, 95% B, 5% C until 2.5 min, followed by an equilibration solution of 60% A, 35% B, 5% C for 2.5 min.

The high-resolution mass spectrometer was operated in full scan mode at 120,000 resolution and mass-to-charge ratio (*m/z*) range 85–1275. Probe temperature, capillary temperature, sweep gas and S-Lens RF levels were maintained at 250 °C, 300 °C, 1 arbitrary units (AU), and 45 AU, respectively, for both polarities. Positive tune settings for sheath gas, auxiliary gas, sweep gas and spray voltage setting were 45 AU, 25 AU and 3.5 kV, respectively; negative settings were 45 AU, 5 AU and -4.0 kV. Raw data files were extracted and aligned using apLCMS^27^ with modifications by xMSanalyzer^28^. Uniquely detected ions consisted of accurate mass *m/z*, retention time and ion abundance, referred to as *m/z* features.

### Statistical analysis

Descriptive statistics were used to evaluate demographics using Student’s t test and chi square where appropriate. Metabolites were first filtered based on coefficient of variation (CV) and Pearson correlation between technical replicates. Only features that have a median CV less than 50% and the samples with Pearson correlation greater than 0.7 are used for further analysis. The technical replicates are averaged following the quality assessment and only features with at least 80% signal in either the rejection group or no rejection group were retained. Metabolite data were then log2 transformed and quantile normalized to reduce the effect of technical errors on downstream statistical analysis and interpretation. Hypothesis testing included one-way repeated measures analysis of variance (ANOVA) using LIMMA via the xmsPANDA R package version 1.0.7.4^6^. The p-values were adjusted for multiple comparisons using Benjamin Hochberg false discovery rate (FDR) procedure. A fivefold cross-validation was used in the OPLS-DA analysis and the mean cross-validation accuracy and corresponding standard deviation were reported. Generalized linear regression was used to compare groups and control for days between biopsy and transplant. In order to explore the direct comparison between ACR and no ACR, we used orthogonal partial least squares discriminant analysis (OPLS-DA) and used a Variable Importance in Projection (VIP) > 2 for further annotation. Type 1 (− log_10_ *p* vs *m/z*) and Type 2 (− log_10_ *p* vs retention time) Manhattan plots were used to visualize the pattern of differential expression across all features with respect to molecular mass and chemical properties, respectively. We conducted univariate linear regression analyses using PLS component scores as the outcome and phenotypes of interest as the independent variables to determine whether the OPLS-DA analysis was successfully identifying those with rejection and without rejection without influence by other factors.

High resolution metabolomics (95 overlapping significant metabolites), clinical data and patient demographics were integrated using xMWAS package in R^29^. Clinical and demographic data included age, height, weight, BMI, biological sex, race, rejection status, liver disease diagnosis, liver enzymes, albumin, total bilirubin, hemoglobin, platelet count, days since transplantation, and steroid use. Integrative network analysis was performed using sparse partial least squares regression analysis, a multivariate approach for data integration that included associations with |r| > 0.4 and p-value < 0.05. The multilevel community detection method in xMWAS was used for identifying communities of tightly connected clinical and demographic data and significant metabolites that differentiated rejection.

### Metabolite annotation

Metabolic features were annotated using xMSannotator in which the confidence scores for annotation are derived from a multi-stage clustering algorithm^30^. Further identification of the selected metabolites were confirmed by criteria of Schymanski et al*.*^31^ either by Level 1 identification, which involves comparing mass spectrum and co-elution relative to authentic standards within a 30-s retention time window, or by Level 2 identification, which involves comparison to METLIN spectral database (http://metlin.scripps.edu/index.php). Lower confidence annotations designated as Level 3–5 identification by Schymanski et al*.*^31^ were made using HMDB (Human Metabolome Database, http://www.hmdb.ca/)^32^ and KEGG (Kyoto Encyclopedia of Genes and Genomes, http://www.genome.jp/kegg/)^33^. Additional manual search was done using METLIN at 5 ppm tolerance^34^. Only metabolites corresponding to Level 1 identification are reported in this manuscript.

Mummichog v2.0 was used to perform pathway enrichment analysis using *m/z* features that were significant at *p* < 0.05^35^. Mummichog was designed to perform pathway and network analysis for untargeted metabolomics. The software compares the enrichment pattern of the significant metabolite subsets with null distribution on known metabolic reactions and pathways, thereby allowing prioritization of pathways for further evaluation^36^. Previously published studies have shown that FDR correction results in type 2 statistical error while protecting for type I statistical error^25^. Pathway enrichment analysis using features significant at raw p-value, provides a 2 step approach which protects against both type I and type II errors^36^.

## Supplementary Information

Supplementary Information.

## Data Availability

Data is provided in the supplementary material.

## Abbreviations

ACR: Acute liver rejection
IPTH: Idiopathic post-transplant chronic hepatitis
DNAIH: De novo autoimmune hepatitis
CT: Computed tomography
MRCPT: Magnetic resonance cholangiopancreatography
MRI: Magnetic resonance imaging
EDTA: Ethylenediaminetetraacetic acid
LC–MS: Liquid chromatography–mass spectrometry
CV: Coefficient of variance
ANOVA: Analysis of variance
FDR: False discovery rate
OPLS-DA: Orthogonal partial least square discriminant analysis
VIP: Variable importance in projection
HMDB: Human metabolome database
KEGG: Kyoto Encyclopedia of Genes and Genomes
GGT: Gamma glutamyltransferase
SD: Standard deviation
AIH: Autoimmune hepatitis
ALF: Acute liver failure
ALT: Alanine aminotransferase
AST: Aspartate aminotransferase
FXR: Farnesoid X receptor
